# An Office-Based Procedure for Hyphema Treatment

**DOI:** 10.1155/2015/321076

**Published:** 2015-03-18

**Authors:** Mikelson MomPremier, Divya Sadhwani, Saad Shaikh

**Affiliations:** ^1^Department of Ophthalmology, Howard University Hospital, Washington, USA; ^2^College of Medicine, University of Central Florida, Orlando, FL 32827, USA; ^3^Orlando Veterans Affairs Medical Center, Orlando, FL 32803, USA; ^4^University of South Florida College of Medicine, Tampa, FL 33612, USA; ^5^Florida State University College of Medicine, Tallahassee, FL 32306, USA; ^6^University of Texas Medical Branch, Galveston, TX 77555, USA

## Abstract

Three cases of hyphema in three different eyes were treated in the office using an anterior chamber fluid-gas exchange technique. Hyphemas were associated with complications of underlying systemic and retinal disease and in the postoperative period of vitrectomy procedures. Patients were successfully treated without requiring return to operating room.

## 1. Introduction

Hyphemas frequently occur as a result of trauma but may also occur as a complication of surgery and spontaneously in certain systemic and ocular conditions. Complications of hyphema include elevated intraocular pressure (IOP), corneal bloodstaining, and glaucoma. Management may range from simple observation, conservative therapy with topical steroid drops, and cycloplegic agents to anterior chamber washout in the operating room [1]. Poor initial visual acuity, glaucoma, vitreous hemorrhage, and eyelid laceration are all associated with long-term poor visual outcome [2, 3].

## 2. Case Series

### 2.1. Case One

A 62-year-old male with a past medical history of human immunodeficiency virus (HIV) infection, uncontrolled diabetes mellitus type II, and prostate cancer currently receiving radiation and chemotherapy presented on postoperative day one after pars plana vitrectomy, fluid-air exchange, and endolaser for retinal detachment repair. He was noted to have a 2.10 mm layered hyphema with diffuse anterior chamber haze from floating red blood cells. One week later the hyphema had worsened layering at 2.75 mm with corneal and anterior capsular bloodstaining. Visual acuity (VA) was hand motion at three feet and intraocular pressure measured 30 mmHg by tonometry. Although an operating room procedure was scheduled, it was cancelled by the anesthesiologist due to an elevated blood sugar level. For this reason, the patient underwent an uncomplicated anterior chamber fluid-gas exchange with 12% perfluoropropane gas (as described below) without complications. One week later, the patient presented with a small gas bubble in the anterior chamber and recurrent diffuse anterior chamber hyphema now accompanied by vitreous hemorrhage. A partially resorbed vitreous gas bubble was noted by ultrasonography with diffuse vitreous hemorrhage layering below. The patient underwent an in-office vitreous fluid-gas exchange followed by a repeat anterior chamber fluid-gas exchange. The patient remained stable without recurrent vitreous hemorrhage or hyphema at follow-up three months later at which time he was scheduled for elective cataract surgery.

### 2.2. Case Two

A 57-year-old male with a past medical history significant for ischemic central retinal vein occlusion (CRVO) in the left eye complicated by retinal and iris neovascularization, hypertension, atrial fibrillation (on coumadin and aspirin), and chronic hepatitis C infection presented with loss of vision secondary to hyphema. Two months prior to presentation, he underwent pars plana vitrectomy, endolaser, and silicone oil injection for recurrent neovascularization and vitreous hemorrhage from central retinal vein occlusion. He was pseudophakic. Visual acuity was hand motion at three feet with an intraocular pressure of 16 mmHg. A diffuse anterior chamber hyphema was present. He underwent an in-office anterior chamber fluid-air exchange followed by anterior chamber bevacizumab (1.25 mg/0.05-cc) injection on the same day. No additional anterior chamber bleeding was noted up until final follow-up 3 months later.

### 2.3. Case Three

A 62-year-old male with a history of chronic hepatitis C infection and liver cirrhosis with thrombocytopenia and hypertension presented in postoperative week one from combined cataract extraction with intraocular lens placement and retinal detachment repair by vitrectomy and gas injection in the right eye. His VA was hand motion at three feet with an intraocular pressure of 38 mmHg. A diffuse anterior chamber hyphema was present with corneal bloodstaining. No vitreous hemorrhage was noted. He underwent on the same day in-office anterior chamber fluid-gas washout with fluid-air exchange. At two-month follow-up no recurrence of anterior chamber hemorrhage was noted.

## 3. Surgical Procedure

**Figure 1:**
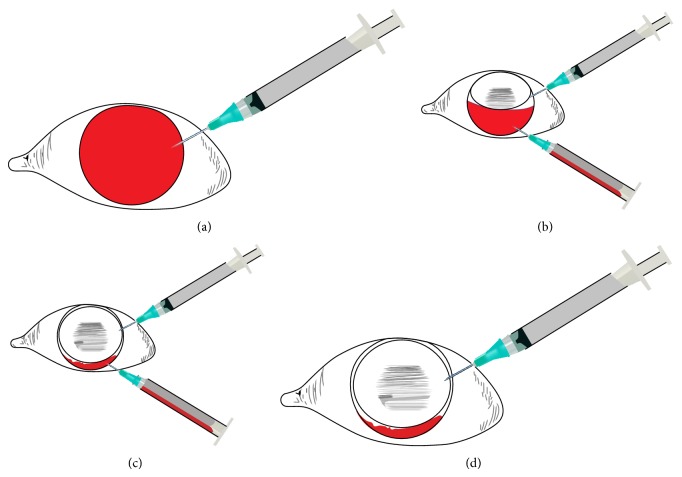
(a) Entry into the anterior chamber superiorly with gas-filled syringe. (b) After partial gas injection, entry into the deepened anterior chamber inferiorly with evacuation syringe, plunger removed. (c) Evacuation of hyphema with complete or near complete anterior chamber fluid-gas exchange. (d) Inferior needle is removed while superior gas-filled syringe is used to equilibrate intraocular pressure.

All patients received a 3 to 4 cc retrobulbar anesthetic injection of 2% lidocaine and 0.75% bupivacaine in a 50 : 50 mixture. All patients were positioned head up in an office examination chair. Thereafter, sterile technique was used including povidone-iodine solution to sterilize the conjunctival surface and lids, sterile gloves, drape, and lid speculum. A 3 cc syringe with filtered gas (12% perfluoropropane or air) with a 27-gauge 1/2 inch needle was inserted through the superotemporal limbal cornea (as close to 12 to 1 o'clock as possible) and into the anterior chamber (Figure 1(a)). Thereafter, a tuberculin (TB) syringe, plunger removed and loaded with a 1/2 inch 27-gauge needle, was inserted into the inferotemporal corneal limbus as close to 5 to 6 o'clock as possible and into the anterior chamber (Figure 1(b)). The tips of each needle were positioned just inside the anterior chamber over the iris to allow for maximal fluid, gas, or air exchange (Figure 1(c)). Gas was injected via the superior syringe while sanguineous anterior chamber fluid was expressed passively through the TB syringe. At the conclusion of the procedure, the TB syringe (bottom) was the first to be removed (Figure 1(d)). The anterior chamber pressure was normalized using the injecting (top) syringe and then removed. All patients were placed on prednisolone acetate 1% and gentamycin ophthalmic solution QID for seven days. Case one underwent a concomitant office-based vitreous fluid-gas exchange using a push-pull method as previously described by Ohana and Blumenkranz [4].

## 4. Discussion

In-office anterior chamber gas injection has been previously described for Descemet stripping automated endothelial keratoplasty (DSAEK) procedures [5]. Similarly, in-office paracentesis of the anterior chamber is a common procedure performed for diagnostic and therapeutic removal of aqueous fluid in the anterior chamber. Currently, most patients are taken to the operating room for surgical management of hyphemas [6]. This report describes how the combination of the above techniques can be used as a quick and effective office-based method in select cases of nonclotted anterior chamber hyphemas.

All three of our patients had preexisting retinal pathology and had recently undergone vitreoretinal surgeries. One patient (case 1) required a repeat of the procedure as well as a vitreous fluid-gas exchange at the time of a second anterior chamber washout procedure. The patients in this small case series in general had multiple systemic comorbidities that predisposed them to spontaneous bleeding, including platelet dysfunction from chemotherapy, chronic hepatic disease, and chronic anticoagulation for atrial fibrillation.

None of the patients required a return trip to the operating room. A disadvantage to the procedure is its limited usefulness in dealing with clotted hyphemas which would not pass through a 27-gauge needle. Another potential limitation is the need to manipulate two instruments at the same time within the anterior chamber. An effective retrobulbar anesthetic injection and case selection against anxious patients who may not tolerate the procedure in an office setting are crucial. Nevertheless, we describe here a quick and effective office-based technique for the management of hyphemas in certain patients.

## References

[1] Walton W., Von Hagen S., Grigorian R., Zarbin M. (2002). Management of traumatic hyphema. *Survey of Ophthalmology*.

[2] Yanoff M. (2009). *Ophthalmology*.

[3] Papaconstantinou D., Georgalas I., Kourtis N. (2009). Contemporary aspects in the prognosis of traumatic hyphemas. *Clinical Ophthalmology*.

[4] Ohana E., Blumenkranz M. S. (1998). Treatment of reopened macular hole after vitrectomy by laser and outpatient fluid-gas exchange. *Ophthalmology*.

[5] Copeland R., Afshari N. (2013). *Principles and Practice of Cornea*.

[6] Simanjuntak G. W. S., Wijaya J., Hasibuan H. (2012). Management of traumatic hyphema with anterior chamber maintainer. *Seminars in Ophthalmology*.

